# Effects of a Board Game on Tic Management and Psychosocial Functioning in Adolescents With Tourette Syndrome: Randomized Controlled Trial

**DOI:** 10.2196/76208

**Published:** 2025-09-09

**Authors:** Mei Yin Lee, Huei Shyong Wang, Chung Yueh Lien, Zhi Hong Chen

**Affiliations:** 1 School of Nursing National Taipei University of Nursing and Health Sciences Taipei City Taiwan; 2 Linkou Chang Gung Memorial Hospital Taoyuan City Taiwan; 3 Department of Information Management National Taipei University of Nursing and Health Sciences Taipei City Taiwan; 4 Graduate Institute of Information and Computer Education National Taiwan Normal University Taipei city Taiwan

**Keywords:** Tourette syndrome, adolescents, board game, mental health, psychosocial functioning

## Abstract

**Background:**

Tics and comorbidities significantly impact the social interactions and mental health of adolescents with Tourette syndrome (TS). Psychoeducation is an initial intervention for TS. Gamification is a common psychoeducational intervention for youths with chronic conditions. However, the effectiveness of board games in improving tic severity and mental health in adolescents with TS remains underexplored.

**Objective:**

We developed a serious board game to investigate its effects on tic severity, mental health, social adjustment, and depression in adolescents with TS.

**Methods:**

A single-blinded, 2-arm, parallel randomized controlled study was conducted. From September 2022 to July 2024, participants were recruited from a medical center in northern Taiwan. Seventy-nine adolescents with TS aged 12 to 18 years were randomly assigned to either a control group (n=39) or an intervention group (n=40). Both groups received care as usual (daily pyridoxine [50 mg] and psychoeducation), while the intervention group additionally participated in a weekly 60-minute board game session over a 4-week period. Outcome measures included the Yale Global Tic Severity Scale, Positive Mental Health Scale, Social Adjustment Scale for Adolescents with TS, and Beck Youth Inventory II – Depression scale.

**Results:**

Generalized estimation equation results showed that, compared to the control group, the intervention group demonstrated significant improvements in positive mental health at the postintervention (β=5.19, 95% CI 0.36 to 10.02, *P*=.04) and follow-up (β=7.14, 95% CI 2.15 to 12.14, *P*=.005), with time-dependent effects. The intervention group also showed significant improvements in social adjustment (β=4.24, 95% CI 1.79 to 6.69, *P*<.001) and depression (β=–3.06, 95% CI –6.04 to –0.11, *P*=.04) at follow-up. No significant differences were observed between the 2 groups in tic severity.

**Conclusions:**

The serious board game developed in this study significantly enhanced psychosocial functioning in adolescents with TS. As an alternative to verbal and written health communication, the board game serves as an innovative psychoeducational instrument for health care professionals to help adolescents with TS in tic management and mental health promotion. Future studies can develop and validate the feasibility of a digital version of the board game.

**Trial Registration:**

ClinicalTrials.gov NCT05566236; https://clinicaltrials.gov/study/NCT05566236

## Introduction

### Background

Tourette syndrome (TS) is a chronic neurodevelopmental disorder characterized by persistent, nonrhythmic motor tics and vocal tics that begin before the age of 18 years [1]. The prevalence of TS in youths is approximately 0.7% higher than in other age groups [2], lighting the need for care services tailored to their specific demands. Around 85% of individuals with TS have at least one psychiatric comorbidity, most commonly attention deficit hyperactivity disorder and obsessive-compulsive disorder [3]. Tics and comorbidities significantly impact the psychosocial functioning [4-7] and mental health of young people [8,9]. Compared to their typical (tic-free) peers, youths with TS exhibit poorer social adjustment [6,7] and higher rates of bullying [10]. Gamified strategies are interventions developed by health care providers to enhance physical and mental health in young people with chronic conditions [11,12]. The effectiveness of board games in improving tic management and psychosocial functioning in adolescents with TS remains unknown.

Due to the stigmatization of TS stemming from social framing and cultural beliefs, young people with TS often feel misunderstood and struggle with interpersonal interactions [9,13,14]. Adolescents with TS who experience higher levels of psychosocial stress demonstrate poorer social adjustment [5]. Depression is a known risk factor for psychosocial maladaptation in adolescents with TS [4]. Mild tics generally do not require treatment if they do not affect a child’s life at school, in social interactions, or at home [15]. However, individuals with mild tics may still face challenges in certain social situations [14]. Psychoeducation serves as an initial intervention, providing patients with information about TS and strategies for coping with it [16]. It also enhances the understanding and fosters positive attitudes among peers, teachers, and family members toward individuals with TS [17-20]. If psychoeducation alone does not meet the care needs of youths with TS, psychological interventions [19] or pharmacological treatments [21] can be considered, provided they are regularly assessed and tailored to the individual needs of the youths [22].

Gamification is one of the psychoeducational interventions developed by health care providers to promote the physical and mental health of youths with chronic conditions. Gamification also transforms the process of imparting health-related knowledge and behaviors into an enjoyable interactive experience [11,12]. Situated learning theory, as a theoretical framework for board game design, incorporates simulation scenarios alongside gamification mechanics and elements to enhance participants’ learning motivation [23,24]. Situated learning theory advocates creating a situated problem-solving context for learners, immersing them in learning. By designing scenarios analogous to learners’ real-life contexts, it facilitates knowledge and skill acquisition through observation and exploratory actions [23-25]. Board games require players to engage in immersive learning scenarios and activities, fostering the development of problem-solving and social skills through discussions or role-playing tasks with other players [11,23,25]. Previous studies have demonstrated the effectiveness of board games in improving care-related skills, knowledge, adjustment, and physical and mental health in young people with chronic conditions [11,26-29]. Two recent studies have shown gamification in behavioral intervention for reducing tic severity [30], but video game elements increase tic frequency [31]. However, whether board games can improve tic and psychosocial functioning in adolescents with TS remains unclear. Parents’ understanding of and attitudes toward TS influence how they educate their children about symptom management [32]. Parental evaluation is especially crucial for adolescents with TS, as it often mirrors the adolescents’ concerns about their future [33]. However, as adolescents grow older, they gradually develop their own strategies for coping with tics and may not always adhere to recommendations from parents or teachers, potentially leading to conflict [13]. Psychoeducation interventions can enhance families’ and school administrators’ understanding of TS, reduce stereotyping [33], and help safeguard the mental health of young people with TS [9,17,22]. Adolescents may also benefit mentally from conventional psychoeducation approaches, including game-based learning [12]. The innovativeness and significance of this study lie in the fact that no previous research has developed a board game–based psychoeducation intervention to improve tic severity and psychosocial functioning in adolescents with TS.

### Aims and Hypotheses

The aim of this study was to examine the effects of a board game intervention on tic severity, mental health, social adjustment, and depression in adolescents with TS compared to the control group. We hypothesized that (1) compared with the control group, implementing the board game intervention would significantly reduce tic severity and depression in adolescents with TS; and (2) compared with the preintervention stage, the intervention group would show improvements in mental health and social adjustment significantly exceeding those of the control group.

## Methods

### Study Design and Procedure

In this single-blind, 2-arm, parallel randomized controlled study, we evaluated the impacts and effectiveness of a board game intervention on tic severity, mental health, social adjustment, and depression in adolescents with TS. In Taiwan, care as usual (CAU) for youths with TS at pediatric outpatient clinics typically includes daily pyridoxine (50 mg) and psychoeducation [34]. Participants were randomly assigned to one of two groups: (1) the intervention group, which received CAU plus a board game intervention, and (2) the control group, which received outpatient CAU only. Changes in participants’ primary outcome measures (tic severity and positive mental health) and secondary outcome measures (social adjustment and depression) were measured at baseline (preintervention, T1), 4 weeks after baseline (postintervention, T2), and 12 weeks after baseline (follow-up, T3). Due to the difficulty and challenging nature of blinding the participants and interveners, only the author responsible for data analysis (CYL) was blinded. This study was registered in the ClinicalTrials.gov registry of the US National Library of Medicine (registration number: NCT05566236).

### Setting and Participants

From September 2022 to July 2024, participants were recruited from a medical center in northern Taiwan and the pediatric neurology outpatient clinics of its branch hospitals. The inclusion criteria were as follows: (1) patients diagnosed with TS [1] by a pediatrician for over a year and aged 12-18 years; (2) patients able to speak and understand Mandarin; and (3) patients willing to participate in the study with parental approval and signed consent. The exclusion criterion was diagnosis with a severe illness, mental illness, or brain injury.

### Sample Size and Randomization

The required sample size was estimated using G*Power 3.1.9.2 statistical software (Heinrich Heine University Düsseldorf), referencing a similar study [34]. With 3 repeated measurements, a Cohen *d* effect size of 0.3**,** a significance level of .05, and a statistical power of 0.80, the required sample size was calculated to be 62. Accounting for a 20 % dropout rate, at least 37 participants were needed in each group.

Randomization and blinding were performed independently by a nonparticipating researcher. Permuted block randomization was conducted using Random Allocation Software 2.0 (Informer Technologies), with 2 groups, a sample size of 92, and a fixed block size of 4. The computer-generated random sequences and group assignments were placed sequentially into tinted envelopes with continuous serial numbers, which were then sealed. After obtaining consent forms from eligible participants, the researcher opened the envelopes in order and assigned participants to either the intervention group or the control group based on the allocation inside the envelope.

### Study Program

#### Intervention Group

**Figure 1:**
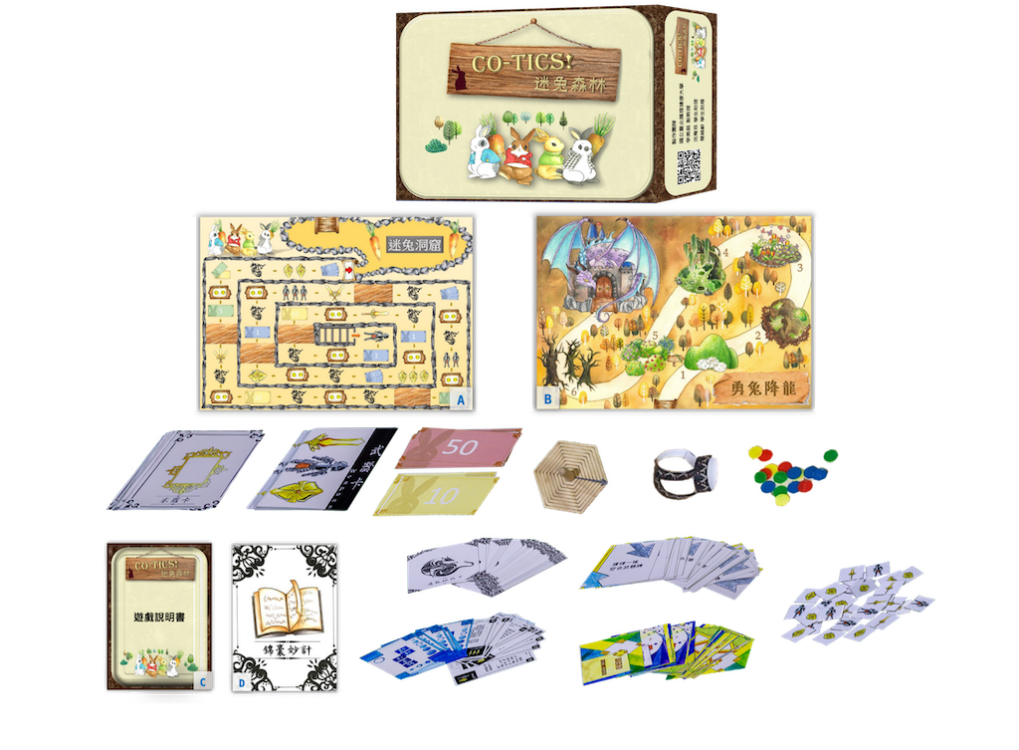
CO-Tics Board Game.

The board game designed in this study, called the CO-Tics Board Game, aimed to encourage adolescents with TS to coexist with their tics through an interactive parent-adolescent gaming experience. To enhance engagement, the game features 2 maps—The Forest of the Lost Rabbit and The Mighty Rabbit Slays the Dragon—along with various game mechanics, such as interactive scenario cards and challenges for acquiring knowledge about TS and accumulating energy coins (see Figure 1). Drawing on insights from our published qualitative studies on youths with TS [9,13] and other studies [20,33], the interactive scenario cards were developed to reflect adolescents’ lived experiences and potential interactions at home, school, and in public settings. The game promotes a nonjudgmental and inclusive attitude among players, encouraging parent and child participants to learn about symptom management while discussing simulated scenarios, sharing their feelings, and exploring coping strategies. The CO-Tics Board Game has acquired a utility model patent (patent number: M636691, applied on January 21, 2023) in Taiwan.

The game sessions were designed to last 1 hour per week over 4 consecutive weeks, with 2 to 3 players per session. The game set includes 2 maps, a game instruction, and a Tips and Tricks Manual, which contains 2 sections: question and answer about TS and interactive scenario card suggestions. Each player starts with 10 energy coins and rolls the dice to determine the order of play. Players roll the dice and move forward. The number on the dice determines the number of squares they can move, and they can take the cards and energy coins indicated in the spaces or move as instructed. There are 45 squares in the Forest of the Lost Rabbit map. The player’s mission is to collect the weapon cards and energy coins needed to complete the Mighty Rabbit Slays the Dragon. There are 6 levels (with 24 interactive scenario cards) in the Mighty Rabbit Slays the Dragon map (Multimedia Appendix 1 provides a more detailed description). First, the player who draws the interactive scenario card is determined by spinning the wheel. After that player responds with their personal thoughts, the other players take turns expressing their opinions, and finally, they can consult the advice in the Tips and Tricks manual for coping strategies for the simulated situation.

The interactive scenario cards address 4 areas: family interactions, school life, social interactions, and coexisting with TS. For example, scenario 1 depicts the following incident:

One time at the bookstore, I was constantly shrugging and clearing my throat. I noticed that the owner kept staring at me and eventually said, ‘You’re acting weird. You should see a doctor if you’re sick.’ I felt hurt by their comment and left the bookstore immediately. I didn’t want to leave the house for a while afterward.

The Tips and Tricks Manual suggests the following for this scenario: some adolescents with TS have experienced judgmental looks from strangers in places like cinemas, restaurants, or buses and have even been directly asked about their unusual movements or sounds. These situations may escalate into conflicts and highlight a widespread lack of understanding about TS. Parents are encouraged to listen to the adolescent’s positive and negative feelings, offering support and validation. To aid players in expressing their emotions about various scenarios, a set of cards was created, featuring 30 types of positive and negative feelings. In addition to the game instructions, we also created a 10-minute video, which can be accessed by scanning a QR code to enhance the participants’ understanding about the gameplay of the board game. We also send weekly reminders through SMS, email, or the LINE app to remind the participants to play the board game for a 60-minute session. We also asked adolescents and their parents to report the time spent playing the game and to submit feedback and questions via a Google Form. These insights allow us to understand and assess the status of the participants’ board game engagement. In this study, the intervention group received CAU and participated in the CO-Tics Board Game to enhance their understanding of TS and develop interpersonal skills and coping strategies.

#### Control Group

The control group (CAU) in this study received daily pyridoxine (50 mg, orally) and at least one paper-based and oral psychoeducation session delivered by a physician or nurse in the outpatient clinic, providing youths with TS and their families with coping strategies for daily life issues caused by tics and comorbidities, such as medication use, reducing tic frequency, and enhancing interpersonal interaction skills, as well as offering psychological support and relevant health care resources.

### Outcomes and Measures

#### Sociodemographic Variables

The demographic information collected for adolescents with TS included age, sex, education level, age at TS diagnosis, comorbidities, and medication use. For their parents, demographic information included age, education level, and marital status.

#### Primary Outcome Measures

Yale Global Tic Severity Scale: The Yale Global Tic Severity Scale (YGTSS) is a widely used instrument for assessing tic severity in adolescents [35]. Items on the YGTSS are scored on a 0-5 Likert scale across 5 dimensions: frequency, intensity, number, complexity, and interference. The YGTSS also includes a total impairment scale that evaluates the patient’s functioning in interpersonal, academic, and occupational domains. The total tic score is calculated by summing the total motor tic score (0-25 points), vocal tic score (0-25 points), and total impairment score (0-50 points), with higher scores indicating greater tic severity. The YGTSS has demonstrated high reliability and validity [36]. The YGTSS scores of the participants in both groups were scored by a pediatric neurologist (a member of our research team).

Positive Mental Health Scale: Adolescence is a critical period for mental health development as it is essential for establishing social interactions and emotional habits [37]. Grounded in positive psychology, Chen [38] developed the Positive Mental Health Scale (PMHS) to assess participants’ mental health status. The PMHS comprises 5 subscales: self-acceptance (3 items), human relationships (5 items), family harmony (6 items), emotional balance (4 items), and optimism and enterprise (7 items). Adolescents self-report their responses, scoring items on a 5-point Likert scale, with a total score range of 25 to 125 points. Higher scores indicate better mental health status. The Cronbach α for the PMHS subscales ranges from 0.76 to 0.92, with a total scale Cronbach α of 0.92. Confirmatory factor analysis has shown the PMHS to have good construct validity and model fit [38].

#### Secondary Outcome Measures

Social Adjustment Scale for Adolescents with Tourette Syndrome: The Social Adjustment Scale for Adolescents with Tourette Syndrome (SASATS) was developed by Lee et al [13] in 2020 based on a qualitative study to measure social adjustment in adolescents. The SASATS is a self-reported measure of an adolescent’s level of adjustment during interpersonal and social interactions over the past 2 weeks. The 17-item SASATS consists of 4 subscales: the relationship between self and TS (4 items), academic performance (5 items), family relationship (5 items), and peer interaction (4 items) [39]. Each item is scored on a scale of 1 to 4 points, yielding a total score range of 17 to 68 points, with higher scores indicating better social adjustment. The SASATS has demonstrated strong psychometric properties, with an α coefficient of 0.87 and a test-retest reliability of 0.81 [39]. A previous study reported a Cronbach α of 0.82 for the SASATS [5].

Beck Youth Inventory II - Depression scale: The Beck Youth Inventory II (BYI-II)- Depression scale evaluates adolescents’ negative thoughts about themselves, life, and the future, as well as feelings of sadness and physiological symptoms of depression [40]. This study used the Chinese version of the BYI-II to measure the self-reported level of depression in adolescents with TS [41]. The scale consists of 20 items scored on a 4-point Likert scale (never, seldom, sometimes, or always), with a total score range of 0 to 60 points. Higher scores indicate more severe depression. The criterion-related validity of the Chinese version is significantly correlated with the original BYI-II. The Chinese version has excellent reliability and validity, with a Cronbach α of 0.93 and a test-retest reliability of 0.81 [41].

### Data Analysis

The data were analyzed using intention-to-treat analysis with SPSS 29.0 software (IBM Corporation). Statistical analysis was conducted at the preparticipation, group assignment, and enrollment stages. Demographic data were analyzed using descriptive statistics, with categorical variables represented as frequency distributions and percentages, and continuous variables represented as means and SDs. The homogeneity of the 2 groups’ preintervention demographic variables and psychosocial functioning was examined using chi-square tests for categorical variables and independent samples *t* tests for continuous variables. For statistical inference, generalized estimating equations were used to analyze differences in social adjustment, positive mental health, depression levels, and tic severity between the 2 groups, over time, and for group-time interactions. The level of statistical significance was set at *P*<.05.

### Ethical Considerations

Ethical approval for the study was obtained from the Institutional Review Board of the Chang Gung Memorial Hospital (201801998B0). The research team strictly adhered to the ethical principles outlined in the Declaration of Helsinki, such as confidentiality, beneficence, respect, impartiality, and nonmaleficence. The researchers explained the study’s purposes and procedures to eligible adolescents and their parents, emphasizing that their rights and interests were protected, that participation was voluntary, and that they could withdraw at any time without providing a reason and without affecting their access to treatment. Participant enrollment commenced only after the adolescents and their parents had signed a consent form.

## Results

### Participant Characteristics

**Figure 2:**
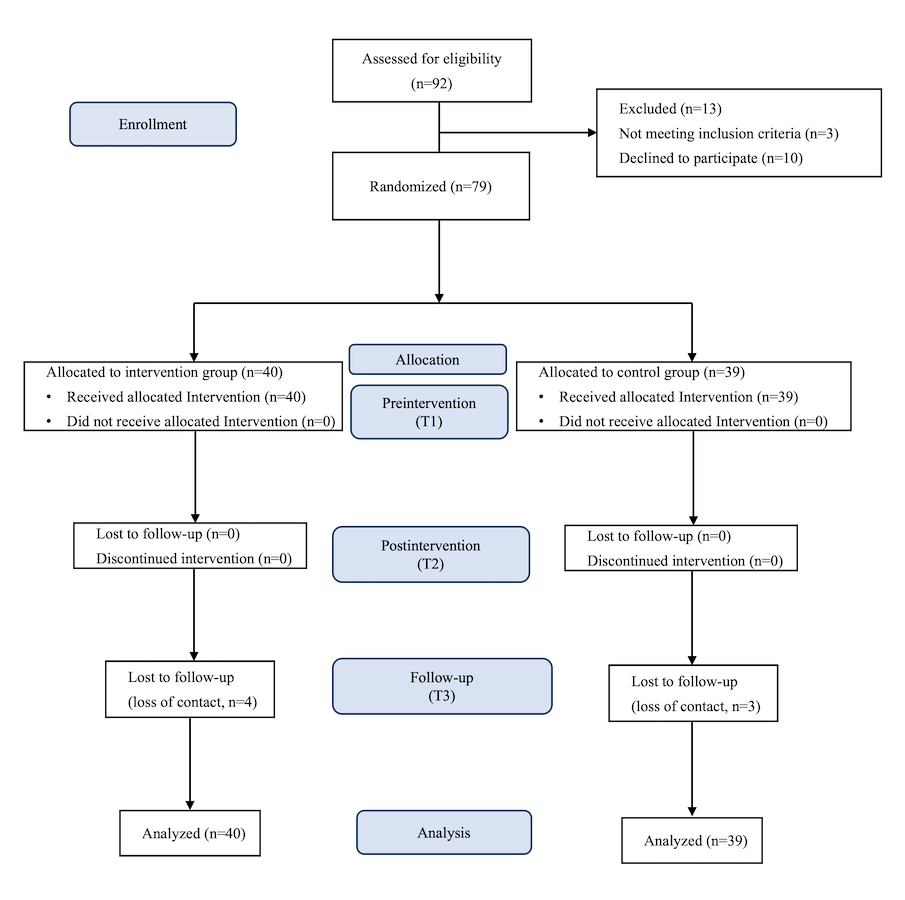
CONSORT (Consolidated Standards of Reporting Trials) flow diagram of participants’ enrolment and recruitment process.

Figure 2 shows the CONSORT (Consolidated Standards of Reporting Trials) flow diagram for the study (Multimedia Appendix 2). Initially, 92 adolescents with TS were screened, and 79 were randomly assigned to either the intervention group (n=40) or the control group (n=39). Seven participants were lost to follow-up, resulting in a dropout rate of 8.9%. A large percentage of the participants were male (n=68, 86%). Approximately 70% (n=56) had at least one comorbidity, primarily attention deficit hyperactivity disorder, and about 32% (n=25) were taking medications, predominantly clonidine. Significant differences were observed between the 2 groups in terms of age (*P*=.002) and education level (*P*=.046), but not for other demographic variables (descriptive statistics for participants’ characteristics are shown in Table 1).

**Table 1:** Characteristics of the participating adolescents and their parents.

Characteristics							Intervention group (n=40)			Control group (n=39)				Chi-square (*df*)				*P* value
**Adolescents**																		
	**Sex**													0.78 (1)				.78^a^
		Male			34			34										
		Female			6			5										
	Age (years), mean (SD)					14.33 (1.66)			15.72 (2.11)				–3.265 (77)				.002^b^	
	**Education level**													6.161 (2)				.046^c^
		Junior high school			31			20										
		Senior high school			7			13										
		University			2			6										
	Age (years) at TS^d^ diagnosis, mean (SD)					7.63 (3.746)			6.33 (4.10)				1.462 (77)				.07^b^	
	**Comorbidity**													0.002 (1)				.96^a^
		No			12			11										
		**Yes**			28			28										
			ADHD^e^, n (%)	18 (46)				21 (54)										
			OCD^f^, n (%)	4 (57)				3 (43)										
			Other^g^, n (%)	10 (59)				7 (41)										
		**Medication intake**											1.284 (1)				.26 ^a^	
		No			25			29										
		**Yes**			15			10										
			Clonidine, n (%)	10 (53)				9 (47)										
			Aripiprazole, n (%)	4 (100)				0 (0)										
			Other^h^, n (%)	6 (75)				2 (25)										
**Parents**																		
	Paternal age (years), mean (SD)					47.85 (4.52)			48.64 (6.81)				–0.609 (77)				.27^b^	
	**Father’s education**													2.344 (3)				.50^c^
		Junior high school			2			0										
		Senior high school			5			7										
		University or higher			33			32										
	Maternal age (years), mean (SD)					45.35 (4.29)			47.15 (6.66)				–1.434 (77)				.08^b^	
	**Mother’s education**													1.353 (3)				.72 ^c^
		Junior high school			1			0										
		Senior high school			7			7										
		University or higher			32			32										
	**Parental marital status**													1.201 (2)				.55^c^
		Married			37			36										
		Divorced			2			3										
		Separated			1			0										

^a^Chi-square test.

^b^Independent *t* tests.

^c^Fisher exact test.

^d^ TS: Tourette syndrome.

^e^ADHD: attention deficit hyperactivity disorder.

^f^OCD: obsessive-compulsive disorder.

^g^Other comorbidities included anxiety and learning disabilities.

^h^Other medications included risperidone and atomoxetine.

### Postintervention Outcomes

All participants in the intervention group completed at least one 60-minute board game session per week for 4 consecutive weeks. No adverse reactions were reported among participants throughout the study period. In the present study, the Cronbach α was 0.65 for the YGTSS, 0.94 for the PMHS, 0.88 for the SASATS, and 0.94 for the BYI-II Depression scale. Table 2 present the mean scores, range and CI for the YGTSS, PMHS, SASATS, and BYI-II Depression scale at the preintervention (baseline), postintervention (4 weeks after baseline), and follow-up (12 weeks after baseline). Additionally, we categorized YGTSS scores as mild (<20) or moderate-to-severe (>20). Chi-square and Fisher exact tests showed no significant differences at baseline (*P*=.31), postintervention (*P*=.31), or follow-up (*P*=.78). The difference in SASATS scores between the 2 groups at follow-up was 4.11 points (*t*=2.25; 95% CI 0.44 to 7.34) with an effect size of 0.53, as shown in Table 2. These results indicate that 8 weeks after completing the board game intervention, the intervention group exhibited a significantly higher level of social adjustment compared to the control group. Table 3 presents the generalized estimating equations results for the outcomes of the board game intervention regarding positive mental health, social adjustment, depression, and tic severity after adjusting for adolescents’ age and education level. There were no significant differences between the 2 groups’ preintervention PMHS, SASATS, BYI-II Depression scale, and YGTSS scores (*P*>.05). Regarding tic severity, no significant differences were observed in YGTSS scores between the 2 groups at either the postintervention (β=–1.5, 95% CI –4 to 1, *P=*.24) or follow-up (β=0.55, 95% CI –2.02 to 3.13, *P=*.68) stages. We further analyzed and found no significant differences in the total tic severity and impairment scores between the 2 groups during the postintervention or follow-up period. The group-time interaction results revealed that, compared to the control group, the intervention group showed significantly greater improvements in PMHS scores at the postintervention (β=5.19, 95% CI 0.36 to 10.02, *P=.*04) and follow-up (β=7.14, 95% CI 2.15 to 12.14, *P*=.005) stages. This suggests that the board game intervention immediately improved positive mental health, with these effects persisting for 8 weeks, indicating time-dependent benefits. The intervention group exhibited significantly more significant improvements in SASATS (β=4.24, 95% CI 1.79 to 6.69, *P*<.001) and BYI-II Depression scale (β=–3.06; 95% CI –6.04 to –0.11, *P=.*04) scores compared to the control group at the follow-up.

We synthesized qualitative information provided via a Google Form by the participants in the intervention group during the board game sessions. Several adolescents reported that the card and map designs were interesting and less rigid and more accessible than standard educational pamphlets or books. Playing the game with a parent not only increased knowledge about TS but also appeared to help parents better understand the disorder’s impact on adolescents’ daily life and social interactions. Some parents indicated that the game facilitated understanding of their child’s thoughts and behaviors; sharing experiences during play enhanced parent-child dialogue and interaction. A few parents further noted that, upon hearing their child’s responses to simulated interpersonal scenarios, they felt both concerned about potential misunderstanding by others and surprised at their child’s more mature coping strategies than they could expect, prompting their reflection on how to adjust their own interactions with their kids.

**Table 2:** Comparison of outcome variables between the intervention and control groups at the baseline (T1), postintervention (T2), and follow-up (T3) time points.

Outcome variables			Intervention group (n=40)					Control group (n=39)					Group difference (95% CI)		Effect size
			Mean (SD)		Range		Mean (SD)			Range					
**Yale Global Tic Severity Scale**															
	T1	15.05 (10.99)		0-52		14.77 (13.27)			0-63		0.103 (–5.17 to 5.73)			0.023	
	T2	14.55 (10.73)		2-50		15.77 (12.69)			0-60		–0.461 (–6.48 to 4.04)			–0.104	
	T3	15.17 (9.94)		3-49		14.05 (10.99)			3-55		0.453 (–3.78 to 6.01)			0.106	
**Total tic severity score**															
	T1	11.05 (6)		0-28		9.36 (6.54)			0-25		1.198 (–1.119 to 4.501)			0.27	
	T2	11.05 (6.36)		2-30		11.67 (7.67)			0-40		–0.39 (–3.769 to 2.536)			–0.088	
	T3	12.11 (6.32)		3-39		10.81 (6.93)			3-35		0.837 (–1.797 to 4.397)			0.196	
**Total impairment score**															
	T1	4 (6.72)		0-30		5.38 (8.22)			0-40		–0.821 (–4.745 to 1.975)			–0.185	
	T2	3.5 (5.8)		0-20		4.1 (6.37)			0-20		–0.44 (–3.33 to 2.125)			–0.099	
	T3	3.06 (5.25)		0-10		3.24 (5.3)			0-20		–0.152 (–2.649 to 2.274)			–0.036	
**Positive Mental Health Scale**															
	T1	93.28 (17.91)		53-124		94.79 (15.42)			40-117		–0.40 (–9.02 to 5.98)			–0.091	
	T2	97.93 (18.58)		52-125		94.26 (15.84)			47-125		0.94 (–4.08 to 11.41)			0.212	
	T3	95.67 (15.44)		63-125		90.36 (14.82)			44-114		1.488 (–1.81 to 12.42)			0.351	
**Social Adjustment Scale for Adolescents with Tourette Syndrome**															
	T1	51.55 (8.54)		25-66		52.36 (6.16)			30-64		–0.48 (–4.15 to 2.53)			–0.108	
	T2	53.75 (7.52)		39-67		53.33 (6.64)			31-68		0.26 (–2.76 to 3.60)			0.059	
	T3	55.42 (7.57)		40-68		51.31 (6.88)			29-67		2.25 (0.44 to 7.34)			0.53	
**Beck Youth Inventory II Depression Scale**															
	T1	13.50 (9.96)		0-36		12.26 (10.69)			0-52		0.54 (–3.38 to 5.87)			0.120	
	T2	13.18 (10.29)		0-46		13.74 (10.27)			0-36		–0.25 (–5.18 to 4.04)			–0.055	
	T3	13.14 (9.99)		0-39		13.94 (10.78)			0-51		–0.33 (–5.69 to 4.08)			–0.078	

**Table 3:** The results of generalized estimating equation on tics severity and psychosocial functions.

Parameters			Estimate (β)		SE		95% CI		Wald chi-square (*df*)		*P* value
**Yale Global Tic Severity Scale^a^**											
	Intercept	8.55		17.17		–25.11 to 42.20		0.25 (1)		.62	
	Intervention group^b^	1.46		2.72		–3.87 to 6.79		0.29 (1)		.59	
	Intervention group × T2^c^	–1.5		1.28		–4 to 1		1.39 (1)		.24	
	Intervention group × T3^c^	0.55		1.31		–2.02 to 3.13		0.18 (1)		.68	
**Total tic severity score^a^**											
	Intercept	25.88		8.77		8.69 to 43.06		8.71 (1)		.003	
	Intervention group^b^	0.26		1.7		–3.08 to 3.59		0.02(1)		.09	
	Intervention group × T2^c^	–2.31		0.78		–3.83 to 0.79		1.82(1)		.10	
	Intervention group × T3^c^	–1.04		1.36		–3.71 to 1.62		0.59 (1)		.44	
**Total impairment** **score^a^**											
	Intercept	0.79		8.66		–16.18 to 17.75		0.008 (1)		.93	
	Intervention group^b^	–0.45		1.49		–3.37 to 2.46		0.093 (1)		.76	
	Intervention group × T2^c^	0.78		0.35		–0.09 to 1.48		2.88 (1)		.08	
	Intervention group × T3^c^	1.34		0.77		–0.16 to 2.84		3.072 (1)		.08	
**Positive mental health scale^a^**											
	Intercept	139.32		23.21		93.82 to 184.81		36.02 (1)		<.001	
	Intervention group^b^	–4.16		3.81		–11.64 to 3.32		1.19 (1)		.28	
	Intervention group × T2^c^	5.19		2.47		0.36 to 10.02		4.44 (1)		.04	
	Intervention group × T3^c^	7.14		2.55		2.15 to 12.14		7.87 (1)		.005	
**Social adjustment scale for adolescents with Tourette syndrome^a^**											
	Intercept	67.28		10.36		46.97 to 87.60		42.15 (1)		<.001	
	Intervention group^b^	–1.44		1.73		–4.82 to 1.95		0.69 (1)		.41	
	Intervention group × T2^c^	1.23		1.21		–1.14 to 3.59		1.03 (1)		.31	
	Intervention group × T3^c^	4.24		1.25		1.79 to 6.69		11.56 (1)		<.001	
**Beck Youth Inventory II** **Depression Scale^a^**											
	Intercept	-6.55		14.83		–35.61 to 22.52		0.19 (1)		.66	
	Intervention group^b^	2.30		2.41		–2.43 to 7.03		0.91 (1)		.34	
	Intervention group × T2^c^	–1.81		1.46		–4.67 to –1.04		1.55 (1)		.21	
	Intervention group × T3^c^	–3.06		1.51		–6.04 to –0.11		4.12 (1)		.04	

^a^Adjusted adolescents’ age and education.

^b^Control group.

^c^Control group × preintervention.

## Discussion

### Principal Findings

Partially in line with our expectations, the board game group’s baseline to postintervention positive mental health, social adjustment, and depression improved significantly compared to the control group. However, no significant changes were observed in tic severity. According to the European clinical guidelines for TS, psychoeducation involves the precise delivery of information about the symptoms, management, prognosis, and daily experience of the condition [17]. It is also a frequently used component in designing comprehensive behavioral interventions for tics [34,42].

### Comparison With Previous Work

Health professionals and researchers often use psychoeducation tools such as written vignettes, videos, workshops, and training programs to provide information about TS [20]. Unlike previous approaches, this is the first study to design a board game intervention based on the psychosocial experiences of adolescents with TS [9,13]. The board game promotes parent-adolescent interactions while addressing the daily life experiences of adolescents with TS and their families. To avoid imbalanced parent-adolescent power dynamics, both players—the adolescent with TS and their parent—begin the game with equal roles, fostering inclusive and nonjudgmental attitudes during gameplay. Mirror cards were specifically designed to enhance parent-adolescent interaction and communication by encouraging both participants to express their feelings about the scenario cards they drew and collaboratively devise coping strategies.

A systematic review found that the duration of board game play in interventions varies significantly, ranging from 5 minutes to 30 hours depending on the study’s objectives and game design [28]. Compared to other studies involving adolescents [11,43], the duration of play in this study was 1 hour per week for 4 consecutive weeks. The 4-week intervention period was chosen because it aligned with the 24 interactive scenario cards designed for the game, allowing for the discussion of 6 different scenarios each week. We found that gamification enhances adolescents’ mental health, consistent with previous studies [11,12,28]. In this study, group-time interaction analysis revealed significantly higher PMHS scores in the intervention group at both the postintervention and follow-up. In other words, the board game enhanced positive mental health in adolescents with TS, demonstrating time-dependent effects. Similar to findings in other studies, board games have been shown to reduce negative emotions in youths with cancer [27] and depression [44]. In the current study, group-time interactions showed that, compared to the control group, the intervention group had significantly lower depression inventory scores, with immediate effects observed at the postintervention stage.

Understanding the social adjustment of youths with chronic illnesses enables health care professionals to develop effective strategies that address their psychosocial functioning and needs [45]. In adolescents with TS, the evaluation of social adjustment includes 4 dimensions: academic performance, family relationships, peer interactions, and the relationship between self and TS [39]. Support from friends, peers, and family plays a critical role in promoting healthy social adjustment in adolescents with TS [13]. An Indonesian study found that a board game intervention reduced stigma, raised awareness of mental health problems, and encouraged positive peer involvement in health-seeking behaviors among senior high school students in remote areas [12]. Similarly, a situated family board game fostered interaction and dialogue between youths with chronic illnesses and their parents, facilitated discussions about daily life, and enhanced self-care skills [26]. In line with these findings, group-time interaction analysis revealed that the intervention group’s SASATS score at follow-up was significantly higher than that of the control group by 4.11 points, indicating that social adjustment improvements were greater 8 weeks after the board game intervention.

Regarding tic severity, the intervention group’s YGTSS score at postintervention was lower by 1.22 point, but no significant group, time, or group-time differences were observed. A possible explanation is because most of the participants had mild tics, and the postintervention follow-up period was only 8 weeks, making it difficult to observe significant long-term changes. Furthermore, in this study, the YGTSS demonstrated a Cronbach α of 0.65, slightly lower than the standard threshold for internal consistency. This may be related to most participants having mild tics and the small sample size; future studies are advised to increase the sample size to potentially improve reliability.

### Strengths

This is the first study to develop a serious board game based on the psychosocial experiences of adolescents with TS [9,13], simulating real-life social interaction contexts to meet their care needs. In addition to enhancing knowledge about TS, the board game also includes a wide array of interactive scenario cards for adolescents with TS and their parents to simulate real-life interpersonal interactions, discuss and share their respective coping methods, and foster parent-child communication. Furthermore, to validate the effectiveness of this intervention, we used several reliable and valid tools to measure the variables related to tic severity and psychosocial functioning. Psychoeducation for TS can be delivered without requiring specialist training in psychotherapy [17]. We recommend that health care professionals consider using the board game as a psychoeducation tool in research and practice to improve psychosocial functioning outcomes in adolescents with TS.

### Limitations and Future Work

Several limitations of this study must be acknowledged. First, the intervention could not be blinded, as participants were required to play the board game. Additionally, performance bias could not be eliminated because the same researcher was responsible for both group allocation and intervention delivery after opening the envelopes. Second, considering that individuals with mild tics also require psychoeducation [14], no tic severity threshold was set as an inclusion criterion, which may have introduced sampling bias. Moreover, this study did not collect data on whether participants in either group experienced changes in medication use during the follow-up period; therefore, the relationship and potential impact of medication on changes in tic severity remain unclear. Third, because the study design was aimed at a parent-adolescent board game and parental feedback was collected via a Google Form, our ability to comprehensively assess parents’ postintervention changes and experiences was limited. Finally, the board game intervention was followed for only 12 weeks, making it impossible to determine its long-term benefits.

Methodological recommendations for future studies are as follows: (1) assign group allocation and intervention delivery to separate personnel to reduce the risk of bias and strengthen internal validity; (2) treat baseline tic severity as an inclusion or stratification criterion to enable subgroup comparisons, and measure and control medication use as a potential confounder; (3) incorporate parental perspectives via focus group interviews or quantitative surveys of parent-child interaction satisfaction, using parental reports as additional indicators of intervention effectiveness; and (4) extend follow-up to 6-12 months to clarify the longer-term effects of the serious board game on adolescents’ psychosocial functioning. We also acknowledge that digital and nondigital board games offer distinct gaming experiences. Future research could develop an interactive digital version of the board game and compare the effects of digital board games, nondigital board games, and CAU on the physical and mental health of adolescents with TS, thereby gaining further insights into the mechanisms and relative effectiveness of different game formats. In addition, the simulated scenarios in this board game were designed specifically for adolescents aged 12-18 years with TS. Future research could adapt the game to the developmental characteristics and life contexts of school-age children, aiming to improve their understanding of TS and reduce potential bullying or stigma among peers.

### Conclusions

In conclusion, this is the first study to develop a serious board game simulating social interaction contexts based on the psychosocial experiences of adolescents with TS. Our findings show that combining CAU with the board game effectively enhances adolescents’ positive mental health and social adjustment and reduces depression, but does not significantly improve tic severity. Our findings support the use of a serious board game to improve psychosocial functioning in adolescents with TS; however, we did not obtain definitive evidence of clinically meaningful reductions in tic severity. To improve the quality of care for youth with TS, future work should develop and rigorously evaluate a digital version of the board game, determining its feasibility and effectiveness in meeting care needs.

## Appendix

Multimedia Appendix 1 Board game summaries.

Multimedia Appendix 2 CONSORT-ehealth checklist.

## Abbreviations

BYI-II: Beck Youth Inventory II
CAU: care as usual
CONSORT: Consolidated Standards of Reporting Trials
PMHS: Positive Mental Health Scale
SASATS: Social Adjustment Scale for Adolescents with Tourette Syndrome
TS: Tourette syndrome
YGTSS: Yale Global Tic Severity Scale

## Data Availability

Data are available from the corresponding author upon reasonable request. The data are not publicly available due to privacy or ethical restrictions.
