# Supplementary material for: Effects of a Board Game on Tic Management and Psychosocial Functioning in Adolescents With Tourette Syndrome: Randomized Controlled Trial
Source: JMIR Serious Games. 2025 Sep 9;13:e76208. doi: 10.2196/76208 (PMC12457861; doi:10.2196/76208)
Supplement: Multimedia Appendix 1 [file games_v13i1e76208_app1.pdf]

**Multimedia Appendix 1 Board Game summaries.**

| Item Name | Descriptions |
|---|---|
| 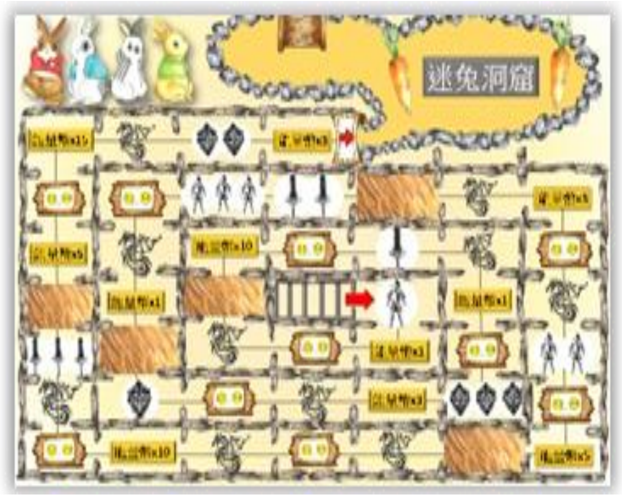<br><br>**Forest of the Lost Rabbit map** | **1.** There are 45 squares in total. The player's mission is to collect the weapon cards and energy coins needed to complete the Mighty Rabbit Slays the Dragon. The Forest of the Lost Rabbit map and cards are briefly described below:<br>**(1)** Weapons: There are 9 weapon squares. Players can get the corresponding Weapon Card indicated by the icon. For example, if a player moves to a square that shows an armor, the player can get a "Faith Armor Weapon Card".<br>**(2)** Energy Coins: There are 11 Energy Coin squares. If the player stops at a square where he/she can get 1 Energy Coin, he/she gets 1 Energy Coin.<br>**(3)** Fortune: There are 10 fortune squares. When the player stops at a "Fortune" square, he/she must draw a Fortune Card (there are 13 types of fortunes) and follow the instructions on the card to complete the mission.<br>**(4)** Flying Dragon: There are 10 flying dragon squares. When the player stops at the "Flying Dragon" square, he/she must draw a Dragon Slaying Tips card and answer the TS question on the card. If a player doesn't know the answer, he or she can consult the Tips and Tricks Manual for the answer at the cost of an energy coin. The player with the correct answer gains 5 energy coins, and the player with the wrong answer loses 3 energy coins.<br>**(5)** Quicksand area: 5 squares in total. When players fall into the quicksand area, they will be unable to move and cannot escape on their own. They can only wait for other players to use PASS cards to rescue them or use FAST PASS cards to save themselves. |

| | |
|---|---|
| 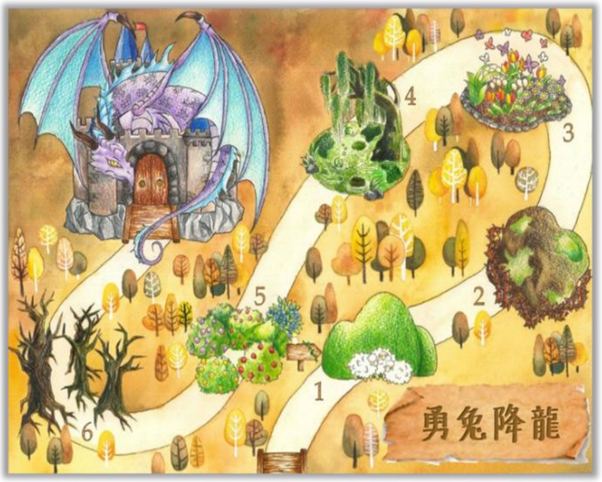<br>**Mighty Rabbit Slays the Dragon Map** | This map has 6 levels (with 24 Interaction Scenario Simulation Cards). First, the player who draws the situation card is determined by spinning the wheel. After that player responds with their personal thoughts, the other players take turns expressing their opinions, and finally, they can consult the advice in the Tips and Tricks manual for coping strategies for the simulated situation. |
| 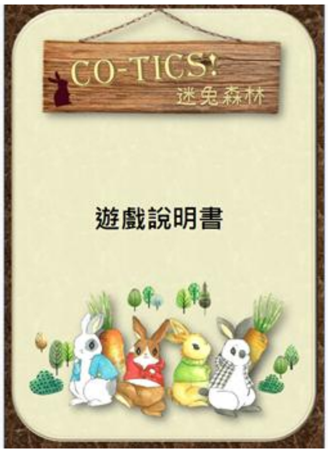<br>**Game instruction** | Each player starts with 10 energy coins and rolls the dice to determine the order of play. Players roll the dice and move forward, the number on the dice determines the number of squares they can move, and they can take the cards and energy coins indicated in the spaces or move as instructed. This board game has two main parts: Part 1 is the " Forest of the Lost Rabbit" map, and Part 2 is the "Mighty Rabbit Slays the Dragon " map. |
| 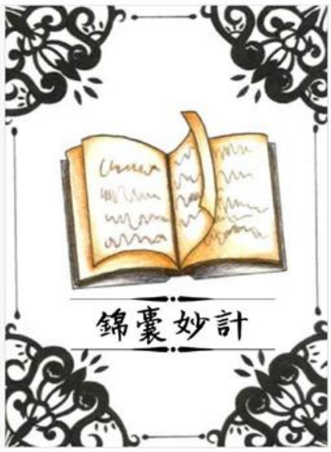<br>**Tips and Tricks Manual** | The Tips and Tricks Manual is divided into two main areas, which are described below.<br>**(1)** Q &A about understanding Tourette Syndrome: There are 20 questions about knowledge of TS and factors that can be aggravators or mitigators of tics. For example, there are 10 Dragon Slaying squares on the Lost Rabbit Forest map where players must answer questions (about Tourette Syndrome) based on the Dragon Slaying card they draw. For example: "Do tics get worse on the first day of school or during big exams?" The answer in the Tips and Tricks booklet is as follows: Patients with severe TS may experience an increase in tic frequency due to changes in new environments, exam stress, and difficulties in interpersonal interactions. However, each individual is unique, and self-observation and recording are required to identify individual environmental factors that exacerbate tics or techniques to alleviate tics.<br>**(2)** Suggestions for the Interaction Scenario Simulation Cards: There are a total of 6 levels in the Cooperative Dragon Slaying Map. Players can randomly draw 6 of the 24 Interaction scenario cards and discuss response strategies together. The |

| | | | |
|---|---|---|---|
| | scenario cards contain simulated scenarios in four major contexts (family interactions, school life, social interactions, and living with TS). The research team refers to relevant literature and clinical care experience to provide suggestions on coping strategies for each situation. Example Scenario 1: “Once in a bookstore, I couldn't help clearing my throat and shrugging my shoulders... I noticed that the owner of the bookstore was staring at me and then said to me, 'You're acting very strangely. If you're sick, you should go see a doctor.' His expression made me feel so bad that I left the bookstore immediately. For a while, I didn't like to go out much." The following advice is provided in the Tips and Tricks manual for the above scenario: Some adolescents with TS have experienced strange stares from strangers in movie theaters, restaurants, buses, etc., and have even been directly asked why they are making strange movements and sounds, which can lead to conflict. These concerns reflect the public's lack of understanding or misunderstanding of TS. It is recommended that family members listen to adolescents with TS and express their support and acceptance of their personal feelings, whether positive or negative. Family members can also work with the teen to role-play how to handle similar situations in the future to avoid hurt or conflict. At the same time, they must respect the teen's wishes about whether to tell others that he or she has TS. In addition, to help players express and describe emotional feelings in a variety of situations, the research team developed the use of mirror cards (containing 30 feelings, such as positive feelings (confidence, courage, etc.) and negative feelings (depression, sadness, helplessness, etc.)). | | |
| **Item Names** | **Descriptions** | **Item Names** | **Descriptions** |
| 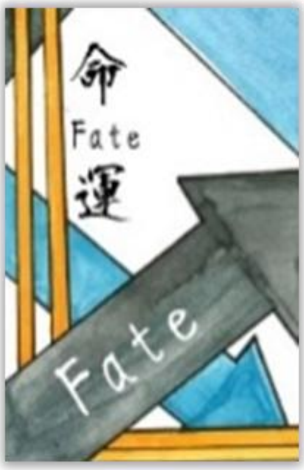  **Fortune Square Cards** | When the player's character lands on this icon, the player must draw a card. There are 13 types of fortunes, such as: winning a prize, sinking in quicksand, returning to the starting point, re-rolling the dice, getting the "I CAN" card, etc. | 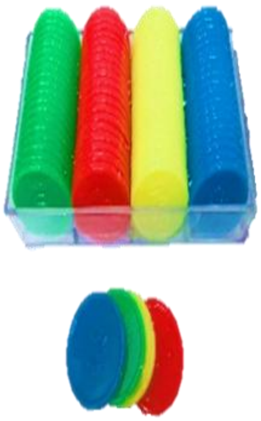 **Energy Coin** | At the start of the game, each player has 10 energy coins, which can be used to purchase items, function cards, etc. |

| | | | |
|---|---|---|---|
| 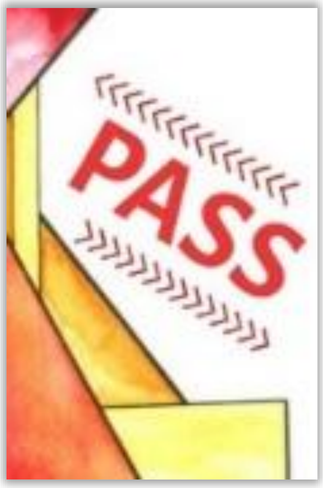  **PASS Cards** | Can only be used once. It allows a player who has fallen into quicksand to escape the quicksand and move three spaces forward. | 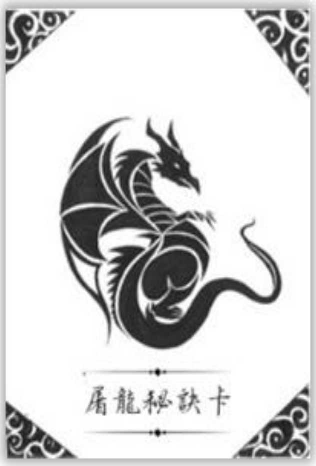  **Flying Dragon Cards** | The player must answer the TS question on the card. The player with the correct answer gains 5 energy coins, and the player with the wrong answer loses 3 energy coins. |
| 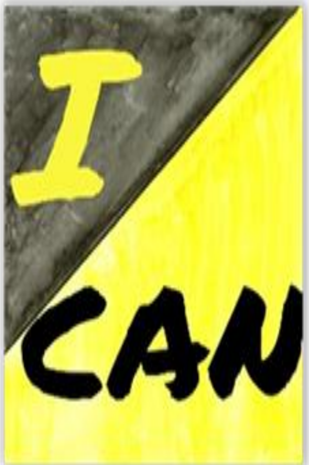  **I CAN Cards** | This is a special ability card. Each player receives one at random before the start of the Brave Rabbit Slaying the Dragon level. It can also be purchased with Energy coins, and its function can be specified. | 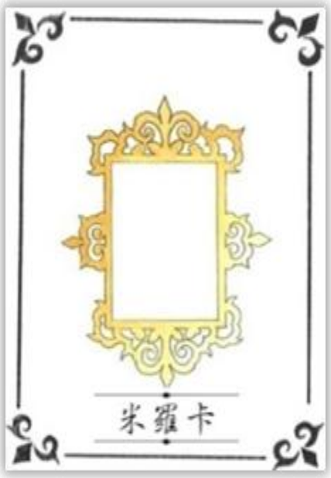  **Mirror Cards** | There are 32 Mirror Cards in total, including 30 feelings, such as positive feelings (confidence, courage, etc.) and negative feelings (depression, sadness, helplessness, etc.), and 2 blank cards. |
| 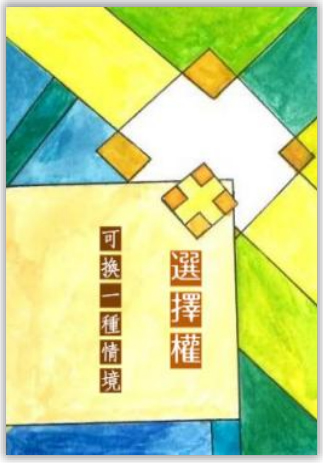  **Option Cards** | You can choose a different scenario card to answer. | 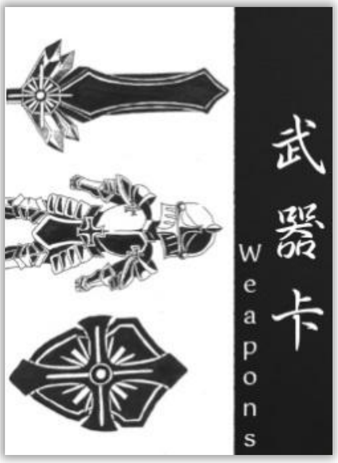  **Weapons Cards** | Players can get the corresponding Weapon Card indicated by the icon. |

| | | | |
|---|---|---|---|
| 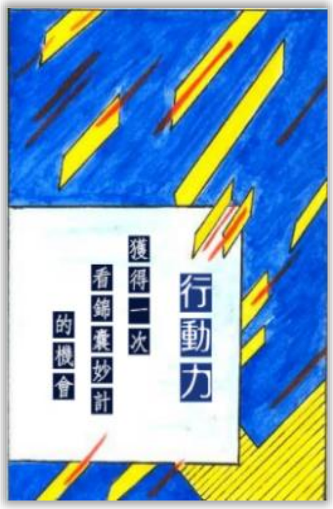<br>**Mobility Cards** | You can access the Tips and Tricks Manual once without spending any energy coins. | 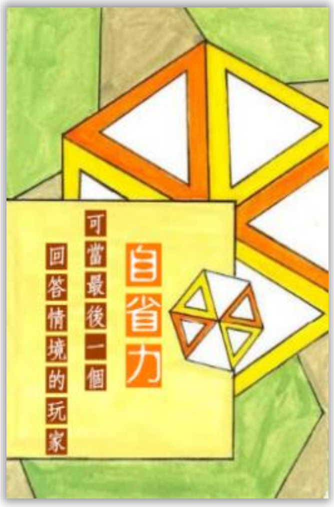<br>**Introversion Cards** | Players have the right to choose the order of answers. |
| 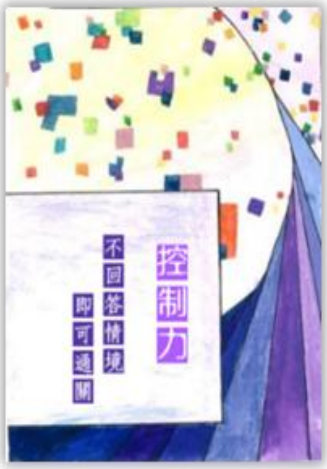<br>**Control Power Cards** | You can unlock the shackles once by not answering. | 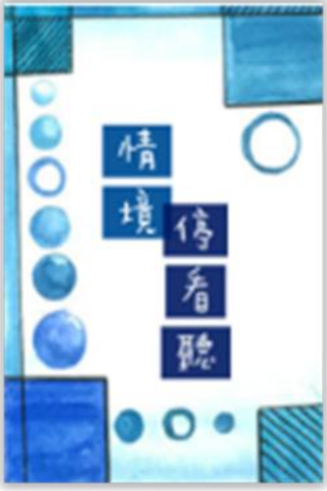<br>**Interactive Scenario Cards** | The cards contain 24 scenarios and address four areas: family interactions, school life, social interactions, and coexisting with TS. |
